# Entry into puberty is reflected in changes in hormone production but not in testicular receptor expression in Atlantic salmon (*Salmo salar*)

**DOI:** 10.1186/s12958-019-0493-8

**Published:** 2019-06-21

**Authors:** Rüdiger W. Schulz, Geir Lasse Taranger, Jan Bogerd, Wouter Nijenhuis, Birgitta Norberg, Rune Male, Eva Andersson

**Affiliations:** 1 Research Group Reproduction and Developmental Biology, Institute of Marine Research, P.O.Box 1870 Nordnes, 5817 Bergen, Norway; 2 Reproductive Biology Group, Division Developmental Biology, Department Biology, Science Faculty, Utrecht University, Utrecht, The Netherlands; 3 Department of Biological Sciences, University of Bergen, Bergen, Norway

**Keywords:** Photoperiod, Puberty, Spermatogenesis, Gonadotropins, Androgens, Growth factors, Hormone receptors

## Abstract

**Background:**

Puberty in male Atlantic salmon in aquaculture can start as early as after the first winter in seawater, stunts growth and entails welfare problems due to the maturation-associated loss of osmoregulation capacity in seawater. A better understanding of the regulation of puberty is the basis for developing improved cultivation approaches that avoid these problems. Our aim here was to identify morphological and molecular markers signaling the initiation of, and potential involvement in, testis maturation.

**Methods:**

In the first experiment, we monitored for the first time in large Atlantic salmon males several reproductive parameters during 17 months including the first reproductive cycle. Since testicular growth accelerated after the Winter solstice, we focused in the second experiment on the 5 months following the winter solstice, exposing fish from February 1 onwards to the natural photoperiod (NL) or to continuous additional light (LL).

**Results:**

In the first experiment, testis weight, plasma androgens and pituitary gonadotropin transcript levels increased with the appearance of type B spermatogonia in the testis, but testicular transcript levels for gonadotropin or androgen receptors did not change while being clearly detectable. In the second experiment, all males kept under NL had been recruited into puberty until June. However, recruitment into puberty was blocked in ~ 40% of the males exposed to LL. The first morphological sign of recruitment was an increased proliferation activity of single spermatogonia and Sertoli cells. Irrespective of the photoperiod, this early sign of testis maturation was accompanied by elevated pituitary *gnrhr4* and *fshb* and testicular *igf3* transcript levels as well as increased plasma androgen levels. The transition into puberty occurred again with stable testicular gonadotropin and androgen receptor transcript levels.

**Conclusions:**

The sensitivity to reproductive hormones is already established before puberty starts and up-regulation of testicular hormone receptor expression is not required to facilitate entry into puberty. The increased availability of receptor ligands, on the other hand, may result from an up-regulation of pituitary Gnrh receptor expression, eventually activating testicular growth factor and sex steroid release and driving germ and Sertoli cell proliferation and differentiation.

**Electronic supplementary material:**

The online version of this article (10.1186/s12958-019-0493-8) contains supplementary material, which is available to authorized users.

## Background

Several aspects of reproductive biology, such as the timing of reproduction, are important for the continuation of a species but likewise for the production of animal protein for human consumption. This also applies to finfish aquaculture and has important consequences for its sustainability [1]. Considering that more than 50% of the fish consumed by humans is derived from aquaculture facilities, possibilities to improve sustainability aspects deserve thorough evaluation. One important aspect in this regard is the timing of puberty. Early puberty, in particular in males, can occur in several species kept under aquaculture conditions, and is often associated with growth retardation or health risks [1]. In a number of economically relevant salmonid species, an additional problem is the puberty-associated loss of the osmoregulation capacity in seawater (where the production sites are situated), eventually resulting in animal welfare problems [2]. Also, reproductively competent fish escaping from aquaculture facilities entail the risk of genetic introgression into wild populations [3]. Hence, we aim at improving our knowledge on the physiological and molecular mechanisms regulating puberty in male fish, which will also help developing approaches for its control and management.

The endocrine control of puberty and adult reproduction involves the integration of external (e.g. photoperiod or temperature) and internal signals (e.g. developmental programs or energy balance) that modulate pituitary gonadotropin release (e.g. [4, 5]). Gonadotropins are required for triggering pubertal testis maturation in all vertebrates. For example, mice with the congenital inability to produce gonadotropin-releasing hormone (GNRH) cannot enter puberty because their pituitary does not release luteinizing hormone (LH) or follicle-stimulating hormone (FSH). However, treatment with FSH [6] or triggering the release of endogenous FSH otherwise [7] stimulated the development of spermatogonia and their entry into meiosis. Recently, near-normal spermatogenesis was reported in the absence of androgens in mice expressing a constitutively active FSH receptor in their Sertoli cells [8]. FSH-driven, androgen-independent proliferation of spermatogonia is also known from primates [9], amphibians [10] and fish [11, 12]. Efficient completion of meiosis and spermiogenesis, on the other hand, is achieved in mammals usually via LH-stimulated androgen signaling [13]. In fish, the requirement for gonadotropic stimulation has been shown by hypophysectomy / gonadotropin replacement studies in European eel, *A. anguilla* [14], and more recently by loss-of-function mutations of pituitary gonadotropin and their cognate receptor genes [15–18] in zebrafish (*Danio rerio*). Different from mammals, both gonadotropins or both cognate receptors need to be inactivated to induce male infertility in fish, reflecting in part overlapping biological activities of the two gonadotropins [19, 20], but also the potent steroidogenic activity of Fsh that is based on *fshr* gene expression by fish Leydig cells [21–23]. Still, Fsh has specific effects in the zebrafish testis that Lh does not have, such as changes in growth factor expression or steroidogenesis-related genes [21]. Fsh-specific effects on testicular gene expression are also reported in rainbow trout (*Oncorhynchus mykiss*) [24, 25]. In fish, androgens stimulate spermatogonial proliferation and differentiation as well as meiosis and spermiogenesis [11, 26–28], while in rodents, androgens are more relevant during meiosis and in particular spermiogenesis but less so during the mitotic phase of spermatogenesis [29, 30]. Overall, FSH and androgen effects on Sertoli cells and other testicular somatic cell types, are the two most relevant endocrine stimuli for pubertal testis maturation and adult spermatogenesis in vertebrates [12, 24, 31–35].

Examining the natural testis development in seasonally reproducing fishes, e.g. sea bass (*Dicentrarchus labrax*) [36], Chinook salmon (*Oncorhynchus tshawytscha*) [37], rainbow trout [38, 39], or Atlantic salmon (*Salmo salar*) parr [40, 41] showed that recruitment into active spermatogenesis was defined as the appearance of type B spermatogonia in many studies, and was associated with increased *fshb* transcript levels and/or Fsh release; testicular *fshr* expression was evident and sometimes reported to increase during recruitment. In the present study, we have investigated recruitment of male Atlantic salmon into puberty in two experiments. First, a complete reproductive cycle was monitored for the first time in large, seawater-adapted Atlantic salmon, analyzing pituitary gonadotropin subunit expression, plasma androgen levels, and expression of testicular gonadotropin and androgen receptors. Our aim was to examine if changes in the testicular sensitivity to gonadotropins or androgens accompany recruitment into puberty. Secondly, focusing on the period after the winter solstice, when recruitment into puberty starts, we made use of the salmon’s sensitivity to photoperiod changes to modulate entry into puberty [42]. In addition to analyzing the parameters mentioned above for the first experiment, we quantified pituitary gonadotropin-releasing hormone receptor gene expression, to investigate changes in the pituitary Gnrh sensitivity potentially associated with pubertal development. In the testis, in addition to gonadotropin and androgen receptor transcripts, we quantified two growth factor (insulin-like growth factor 3 (*igf3*) and anti-Mullerian hormone (*amh*)) transcripts that responded in opposite manners to androgen-mediated stimulation of spermatogenesis in immature salmon [43], played antagonistic roles in spermatogenesis [11, 44] and were regulated in opposite directions by Fsh in zebrafish [45]. Histological analysis of germ cell development formed the basis for both experiments.

## Methods

### Experimental animals

Atlantic salmon of AquaGen origin (AquaGen AS, Trondheim, Norway) were hatched and reared in freshwater indoor facilities at the Institute of Marine Research, the Matre Aquaculture Research Station, Matredal (61°N), Norway. The experimental groups were subjected to rearing conditions that were similar to standard commercial fish farming conditions. Such conditions are listed as an exception in The Norwegian Regulation on Animal Experimentation, so that approval of the experimental protocol of this experiment by the Norwegian Animal Research Authority (NARA) was not needed (http://www.mattilsynet.no/dyr_og_dyrehold/dyrevelferd/forsoksdyr/). Blood sampling and decapitation immediately thereafter occurred under anesthesia (see below). All experiments were done at the Institute of Marine Research, Matre Research Station which is authorized for animal experimentation (Norwegian Food Safety Authority, facility 110) and in accordance with International guidelines.

### Experiment 1. Normal pubertal development in Atlantic salmon

The fish were transferred from freshwater indoor facilities to sea cages after smoltification at 18 months of age. These previously immature fish (*n* = 950, mean body weight; 6.3 kg; some individuals showing signs of previous maturation were excluded from the experiment) were maintained under natural light, initially in a sea cage of 12 × 12 × 12 m, later on from January in smaller sea cages of 5 × 5 × 8 m (l x w x d). Sampling commenced in August when the fish had been 15 months in seawater. The fish were sampled on 15 occasions, the first 3 ones bimonthly (i.e. August, October and December), the subsequent 12 ones monthly from January to December. Hence, the sampling covered a 17 months-long period. During this period, the fish were kept in seawater from August until May. After this last sampling in seawater, 160 randomly selected fish (due to space limitations, not all fish could be transferred to freshwater) were transferred to two 5 m indoor circular freshwater tanks (19.6 m^3^ each) with simulated natural photoperiod (61°N) and ambient water temperature. This transfer mimicked the natural salinity change salmon experience during their spawning migration. The sampling continued monthly in freshwater and ended in December.

In the sea cages the fish were fed Bio-optimal dry feed (BioMar LTD, Trondheim, Norway) until satiation using a computer-operated feeding system. After transfer to the indoor freshwater tanks there was no feeding, mimicking the natural cease of feeding in salmon during their spawning migration.

### Experiment 2. Initiation of puberty under natural or additional continuous light

Previously immature Atlantic salmon (*n* = 362, mean body weight: 5.8 kg; some individuals showing signs of previous maturation were excluded from the experiment) were maintained as described for the fish in experiment 1 until January (18 months in sea water). The fish were distributed randomly over two sea cages (5 × 5 × 8 m, l x w x d), with approximately 170 fish in each cage. One cage was exposed to natural light throughout (NL group). The second cage was exposed to continuous additional light (LL group) from February 1 until June 11. The light was supplied by a single 400 W metal halogen bulb mounted 1 m above the water surface on the edge of the cage. The fish were fed as described above. An initial control sample was collected on January 8. Further samples were collected at 4 time points: February 18, March 19, April 25, and on June 11, when the experiment was terminated.

### Sampling

At each sampling, the fish were netted from the cages, immediately anaesthetized with 6 ppt metomidate (Syndel, Victoria, B.C.) and weighed (total body weight). Blood (~ 5 ml) was collected in heparinized syringes from the caudal vein. After decapitation, pituitary and gonad tissues were excised. The pituitaries were wrapped in aluminum foil and snap-frozen in liquid nitrogen. Gonads were weighed to calculate the gonado-somatic index (GSI = gonad weight (g) *100 / total body weight (g)) and transversally cut fragments of testis tissue from the cranial third of the organ were fixed in phosphate-buffered 4% paraformaldehyde for in situ hybridization purposes, in Bouin’s fixative for routine histological analysis, or snap frozen as described above for pituitary tissue. All frozen tissues were stored in − 80 °C until further analysis.

### Real-time, quantitative PCR assays

RNA isolation, cDNA synthesis and subsequent real-time quantitative PCR (qPCR) was carried out as described in detail previously [46], except for the RNA isolation from the pituitaries from experiment 1, where the PARIS kit (Ambion) was used, as described previously [47]. All qPCR assays were performed in duplicate, using 96-well optical plates on an ABI Prism Sequence Detection System (Applied Biosystems) using default settings.

In the pituitary, transcript levels of Atlantic salmon *fshb* and *lhb* were quantified as described in [42], and of *gnrhr4* as described in [43]. In testis tissue, transcript levels of *fshr*, *lhcgr* and the endogenous control (elongation factor 1α, *ef1α*, also determined in the pituitary) were quantified as described in [46], those of the two growth factors *igf3* and *amh* as described in [48]. Finally, the primer sequences and characterization of the qPCR assays for the two androgen receptors genes *ara1* and *ara2* are given in Table 1. Expression levels were calculated relative to the initial values in August in experiment 1, and relative to the initial values in January in experiment 2. To calculate gene expression data the ΔΔCt method was used, as described previously [49].

**Table 1:** Forward and reverse primers, and TaqMan fluorogenic (FAM-TAMRA or FAM-MGBNFQ) probes, used in real-time, quantitative PCR assays to determine the relative *ara1* and *ara2* mRNA levels in Atlantic salmon.

Target	GenBank accession no.	Primer^a^	Sequence (5′➔3′)^a^	Slope^b^
*ara1*	KJ584693	*ara1*-Fw	CGCAGAATTGCCCTTAAGCA	−3,54
		*ara1*-Rv	CCACCATGAAGGCGAAAATG	
		*ara1*-probe	FAM-CACAGACAACAGGGAAAAATAGCCACGATT-TAMRA	
*ara2*	KJ584694	*ara2*-Fw	TGTCGCTCTGGACCGTTAGC	−3,60
		*ara2*-Rv	ATGGTGGTGCAGGCAGAATT	
		*ara2*-probe	FAM-CAGTGCTGTCAACCAGCTGTGCCC-TAMRA	

^a^Sequences are shown for the sense (Fw) and antisense (Rv) primers, and the TaqMan probe (probe). Probes have a 6-FAM label at their 5′-ends, and a TAMRA quencher label at their 3′-ends

^b^Slope for primer/probe combinations obtained in validation experiments using four 10-fold dilutions of testis cDNA

### Testis histology, proliferation analysis and in situ hybridization

Bouin-fixed gonads were dehydrated, embedded in paraffin, sectioned at 5 μm and stained with hematoxylin/eosin (in some cases in combination with periodic acid Schiff), to evaluate the progress of spermatogenesis. We distinguished undifferentiated type A spermatogonia (A_und_; single cells with no or very little heterochromatin in the nucleus, one or two prominent nucleoli and a nuclear diameter of ~ 13 μm) and differentiating type A spermatogonia (A_diff_; two or more [usually not more than 3 or 4 visible] cells in a spermatogenic cyst with nuclei still containing little heterochromatin, and one or two nucleoli but with a smaller nuclear diameter of ~ 9 μm). Type B spermatogonia formed larger cysts with many cells, showing a smaller nuclear diameter, no discernible nucleoli, but ample heterochromatin. Moreover, different from the type A spermatogonia where nuclei usually were round, the nuclei in B spermatogonia often were laterally compressed and appeared elongated. Since the nuclei in B spermatogonia were surrounded by only a thin layer of cytoplasm, the nuclei appeared densely packed in their cysts. Spermatocytes (SC) showed again an increase in nuclear diameter compared to B spermatogonia, the nucleus was rounded again and contained meiotic chromosomes in different stages of condensation, while the cytoplasm remained nearly unstained. Finally, we identified spermatids (ST) that are still situated within the lumen of spermatogenic cysts, and spermatozoa (SZ) free in the lumen of spermatogenic tubules after spermiation, i.e. opening of the spermatogenic cysts.

In experiment 2, 35 testis samples showing type A spermatogonia as furthest developed germ cell type were found in the two photoperiod treatment groups NL and LL. These samples were analyzed for proliferation activity, using an antibody against phosphorylated histone H3 (pH 3). Immunohistochemistry and quantification of proliferation was carried out as described previously [43], except that digital images were taken at a magnification of 400x (instead of 1000x), and that 10 (instead of 25) viewfields were analyzed per individual. Results are expressed as average number of pH 3-positive cells per viewfield.

In situ hybridization (ISH) experiments served to localize *amh* mRNA. Five μm thick sections from paraffin-embedded, paraformaldehyde-fixed testis tissue were used from selected animals showing either low or high proliferation activity and plasma androgen levels. The cRNA probes for ISH were produced using 841 bp salmon *amh* cDNA (from nts 827 to 1667, accession number AY722411) inserted into pCR4-TOPO plasmid. The plasmid templates were linearized with *Not* I and *Spe* I, allowing for sense (with T3 polymerase) and antisense (with T7 polymerase) probes, respectively, to be synthesized. Probe synthesis and ISH were subsequently carried out as described previously [50], except; a) the post-fixation after rehydration was omitted, b) section permeabilization time with proteinase K was increased to 20 min; and c) the probe concentration was increased to 2 μg/ml of hybridization solution.

### Measurement of plasma androgens

Testosterone (T) and 11-ketotestosterone (11KT) were extracted from individual plasma samples and quantified by enzyme-linked immunosorbent assay (ELISA) as described previously for 11KT [51] and T [52], respectively. Minor modifications were applied, exactly as described in [53].

### Statistical analyses

Data were analyzed by Statistica 10 (StatSoft Inc., USA). Differences in reproductive parameters across different histological stages and light treatments were assessed by analysis of variance (one way ANOVA) on log-transformed values, followed by Student Newman Keuls (SNK) post hoc test on data grouped by histological classification comparing all germ cell stages with each other. Data were checked for homogeneity of variance by Levene’s test and for normal distribution using normal plots. Two group comparisons were done with Student t-test. In all cases, the results are presented as mean and their standard errors; the number of records per group are given in the figures.

Because of the loss of some pituitary samples and because of insufficient sample quality in a number of cases when testis tissue contained a high proportion of haploid cells, the number of records can be lower for the gene expression data than for GSI and sex steroid plasma levels in the same groups. In experiment 2, one subgroup (pituitary expression data from LL-exposed animals with spermatogenesis progressed to meiotic/postmeiotic cells) consisted of two males only. The data of these two males are shown as data points in Figs. 5 and 7 but were excluded from the statistical analyses.

## Results

### Experiment 1. Normal pubertal development

Testis samples were analyzed histologically and each male was assigned to a developmental stage depending on the presence of the furthest developed germ cell type (A_und_, A_diff_, B, SC/ST, or SZ). Since fish at two different stages were identified on all sampling dates (Additional file 3: Table S1), we present the data based on the progress through the stages of germ cell development rather than based on sampling date.

**Fig. 1:**
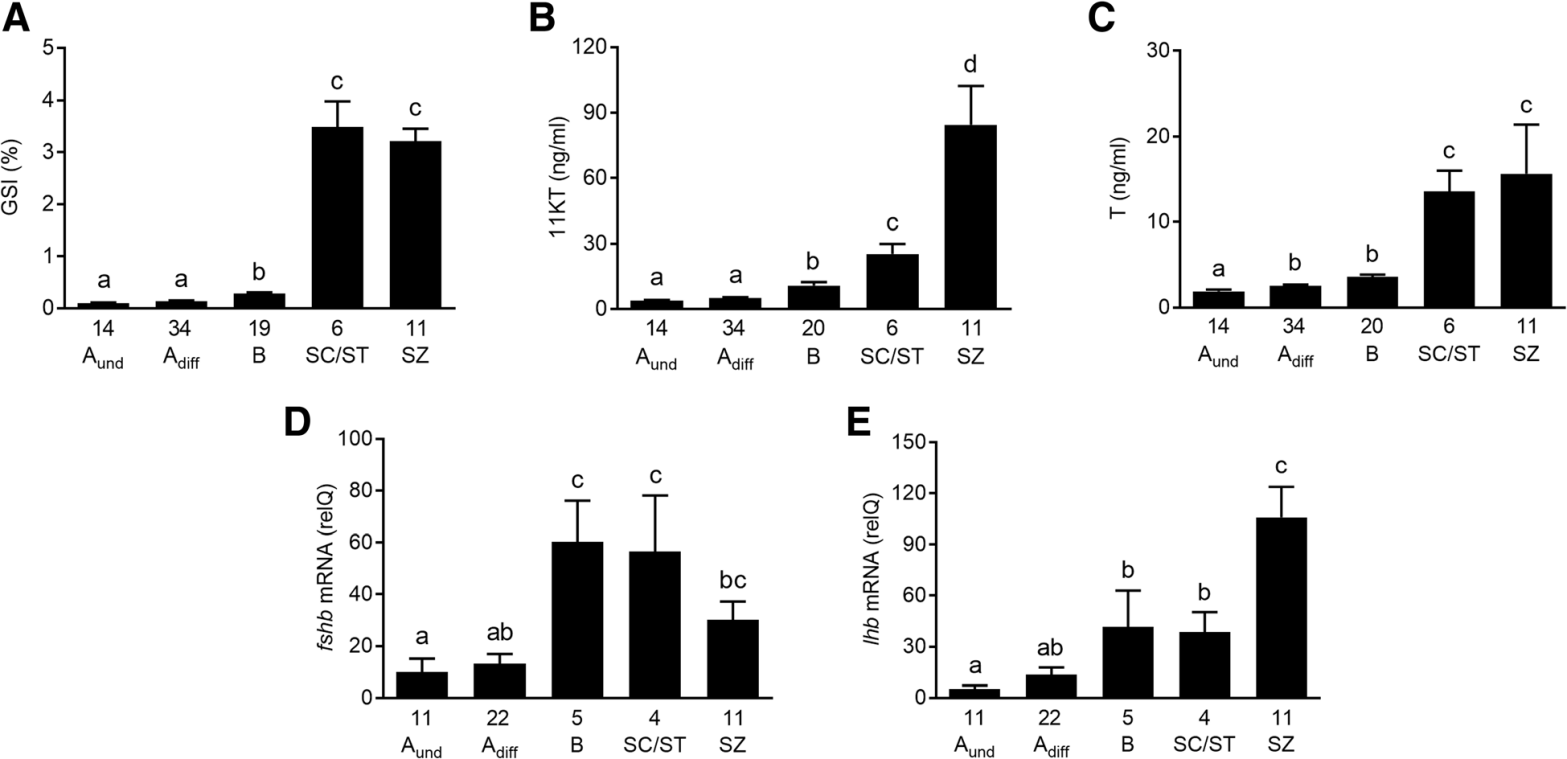
Reproductive parameters in Atlantic salmon male grilse during their first reproductive cycle. Gonadosomatic index (GSI; **a** plasma 11-ketotestosterone (11KT; **b** or testosterone (T; **c** levels (ng/ml plasma), and relative pituitary mRNA levels of *fshb* (**d**) or *lhb* (**e**) in male Atlantic salmon. Samples were collected over a period of 17 months during the first reproductive cycle of these 3 year old males (experiment 1). The animals were grouped according to the histological analysis of the most advanced germ cell stage found in the testis (A_und_, type A undifferentiated spermatogonia; A_diff_, type A differentiating spermatogonia; B, type B spermatogonia; SC/ST, spermatocytes/spermatids; SZ, spermatozoa). The number of individuals analyzed per group is given under the respective bars, showing group means and SEM. Bars sharing the same letter within a given graph are not significantly different (*p* < 0.05; one way ANOVA followed by SNK post hoc test)

The first significant increase in GSI occurred when type B spermatogonia were found (Fig. 1a), followed by a major increase associated with the appearance of meiotic and postmeiotic germ cells.

Similar to the GSI data, the first significant increase in 11KT plasma levels was associated with the transition to type B spermatogonia (Fig. 1b). The values kept increasing significantly with the progress through spermatogenesis (Fig. 1b). However, the most prominent increase to maximum levels of ~ 80 ng/ml took place when spermatogenesis approached completion.

Gradual increases in plasma T levels were recorded during the different spermatogonial stages (Fig. 1c). Different from 11KT, however, T concentrations reached a high plateau already with the appearance of meiotic cells, and T levels did not increase further when spermatozoa appeared in the testis (Fig. 1c). In general, plasma T levels were 3–5 times lower than those of 11KT.

Pituitary *fshb* transcript levels were clearly up-regulated in association with the appearance of type B spermatogonia (Fig. 1d). High levels were maintained when meiotic cells were formed. The *fshb* transcript levels started to decline towards the completion of spermatogenesis.

Pituitary *lhb* transcript levels increased as well when type B spermatogonia appeared in the testes, although less prominently than was seen with the *fshb* transcript. In contrast to the latter, peak levels of *lhb* transcripts were reached when spermatogenesis approached completion (Fig. 1e).

**Fig. 2:**
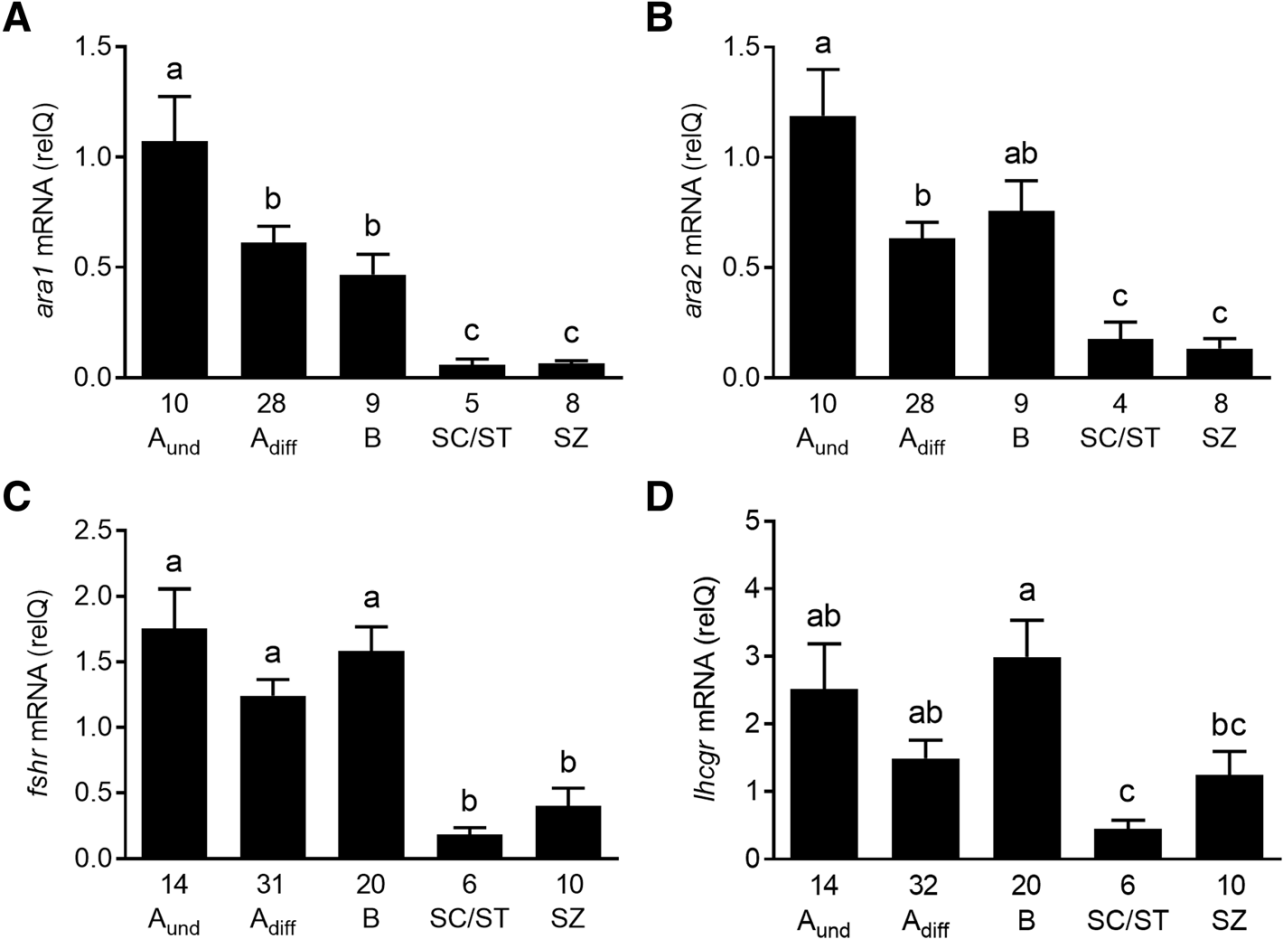
Testicular mRNA levels of androgen and gonadotropin receptors during the first reproductive cycle of Atlantic salmon grilse. Relative mRNA levels of *ara1* (**a**) or *ara2* (**b**) and of *fshr* (**c**) or *lhcgr* (**d**) are shown. Samples were collected over a period of 17 months during the first reproductive cycle of these 3 year old males (experiment 1). The animals were grouped according to the histological analysis of the most advanced germ cell stage found in the testis (A_und_, type A undifferentiated spermatogonia; A_diff_, type A differentiating spermatogonia; B, type B spermatogonia; SC/ST, spermatocytes/spermatids; SZ, spermatozoa). The number of individuals analyzed per group is given under the respective bars, showing group means and SEM. Bars sharing the same letter within a given graph are not significantly different (*p* < 0.05; one way ANOVA followed by SNK post hoc test)

Regarding testicular receptor gene expression, all four transcripts analyzed (*ara1*, *ara2*, *fshr*, *lhcgr*) were clearly detectable in immature males (Fig. 2). In general, higher levels were recorded during the spermatogonial stages when GSI levels were low, while lower levels were characteristic of stages displaying elevated GSI levels towards the meiotic/postmeiotic stages (Fig. 2a-d). In addition to this general trend, we noted that androgen but not gonadotropin receptor transcripts showed a significant decrease associated with the transition from A_und_ to A_diff_ spermatogonia. We therefore analyzed in more detail this transition period, characterized by the presence of two stages of spermatogenic development (type A_und_ and A_diff_ spermatogonia) that were found over a period of ~ 8 months (see Additional file 3: Table S1).

**Fig. 3:**
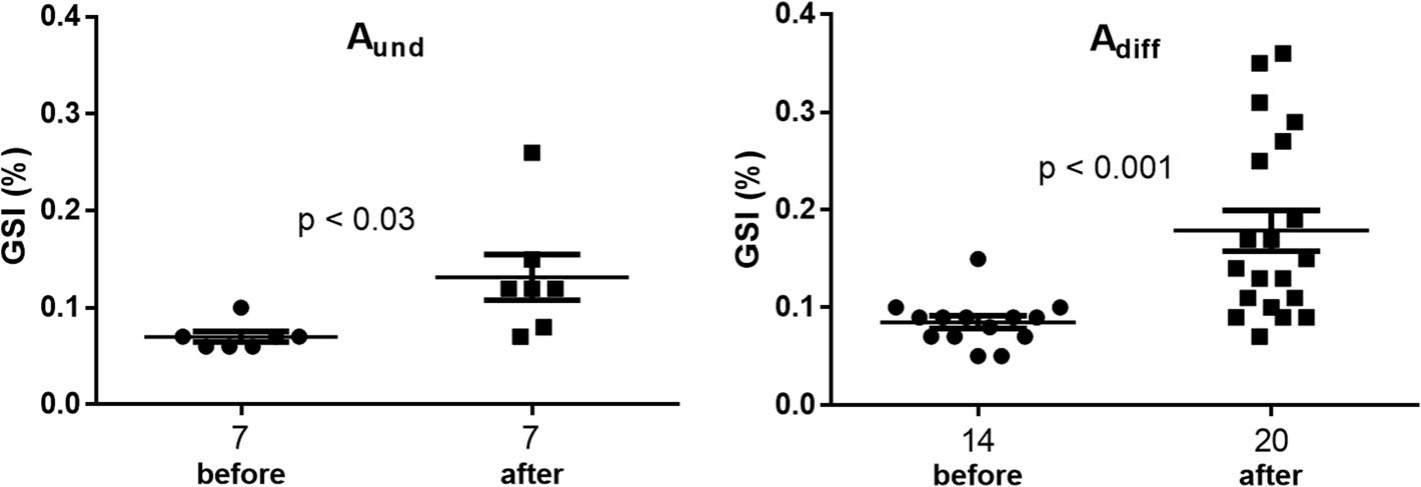
GSI values of Atlantic salmon males showing type A_und_ or type A_diff_ spermatogonia as furthest developed germ cell type, sampled before or after the Winter solstice. Individual values, mean and standard error of the mean are shown. The number of individuals analyzed per group is given under the respective groups. Means were compared by Student t-test

When comparing males showing type A spermatogonia that were sampled before vs. after the winter solstice, significantly higher GSI levels, frequently above 0.1%, were found in males sampled after the winter solstice (Fig. 3). Higher levels after the winter solstice were also observed for pituitary *fshb* mRNA and plasma 11KT in males with type A_diff_ spermatogonia (Additional file 1: Figure S1). This observation allows two suggestions. First, considering that A_diff_ were found during several months before the winter solstice (Additional file 3: Table S1), the transition from type A_und_ to type A_diff_ spermatogonia apparently takes place at a low rate during the allometric growth of the testis and does not reflect a specific endocrine input triggering this step of germ cell development. Second, the increased production of both, A_und_ and A_diff_ spermatogonia that started after the winter solstice, as reflected in the superallometric growth of the testis, opens the possibility that a photoperiod-triggered signal recruited type A spermatogonia into an elevated level of proliferation activity.

To further our understanding of this developmental step, apparently associated with the increasing daylength, we focused in the second study on the period after the winter solstice, including an experimental photoperiod condition into the study design.

### Experiment 2. Puberty under natural or continuous light

**Fig. 4:**
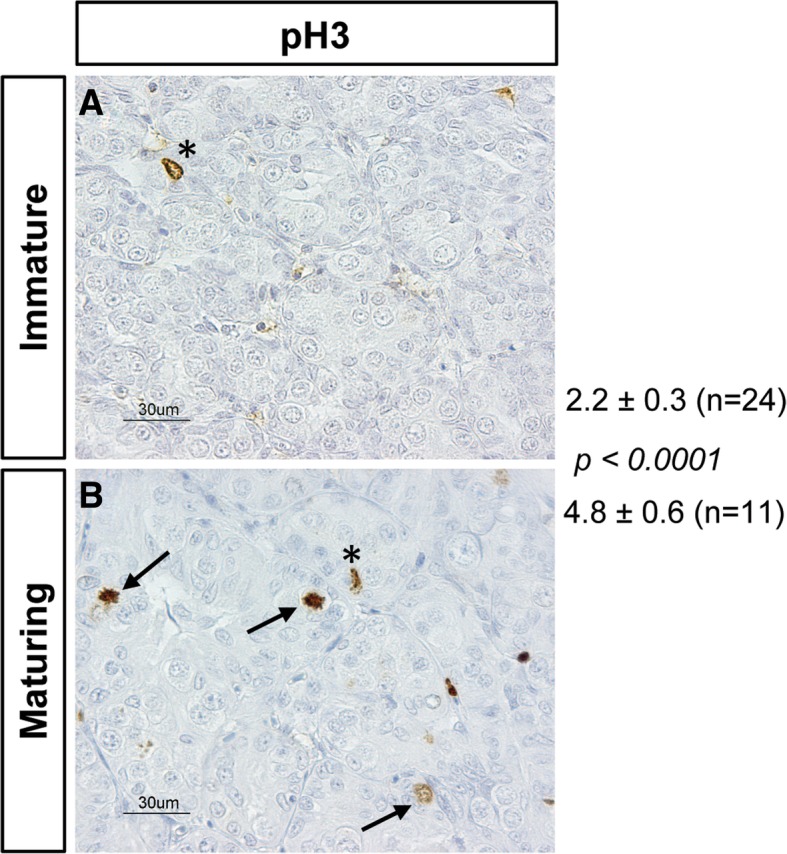
Immunocytochemical detection of proliferation activity in Atlantic salmon testis. The proliferation marker phosphorylated histone H3 (pH 3; brown staining in nuclei) and hematoxylin counterstain (light blue) of testis sections from males of experiment 2 in the SGA stage. pH 3-positive germ cell (arrows) and Sertoli cell (asterisks) nuclei were counted; pH 3 staining in the interstitial area was disregarded. Males in the SGA stage showed a low (**a**; immature; *n* = 24) or an elevated (**b**; maturing; *n* = 11) number of pH 3-positive nuclei (mean ± S.E.M. per viewfield at 400-fold magnification) that differed significantly (*p* < 0.0001; Student t-test). Bars indicate 30 μm

The males in experiment 2 were grouped according to the histologically determined stage of germ cell development and photoperiod treatment (NL or LL) as regards individuals showing type B spermatogonia (SGB) or spermatocytes/spermatids (SC/ST) as the furthest developed germ cells; all these males had been recruited into pubertal development. However, the group of males showing type A spermatogonia (SGA) as the furthest developed germ cell type, contained individuals collected during the January sampling, when LL treatment had not started yet, and males from all later samplings and both photoperiod groups (see Additional file 4: Table S2). Therefore, males in the SGA stage were grouped based on being recruited into puberty or not, which was determined by analyzing the proliferation activity in the testis. Immunocytochemical detection of the proliferation marker pH 3 showed that the 35 males showing SGA as furthest developed germ cell type can be grouped into individuals showing a low (*n* = 24) or a high (*n* = 11) proliferation activity (Fig. 4). In most individuals (all immature males and 7 out of 11 maturing males), proliferation of single cells was observed, being either germ or Sertoli cells (Fig. 4), while in the remaining 4 individuals, also pairs or groups of 3 or 4 cells showed pH 3-positive nuclei.

Fish exposed to NL conditions were progressively recruited into maturation such that by the last sampling in June, immature males were no longer encountered, and all males had reached meiosis, as indicated by the presence of SC in the testes. Exposure to continuous light, in contrast, resulted in a split of the population. Under LL conditions, in about 40% of the males found at all sampling dates (Additional file 4: Table S2), entry into puberty was blocked and the fish remained immature. On the other hand, about 60% of the males were maturing, and in June, half of them had not only reached meiosis, as the males under NL conditions, but had progressed into spermiogenesis, i.e. spermatids were found.

**Fig. 5:**
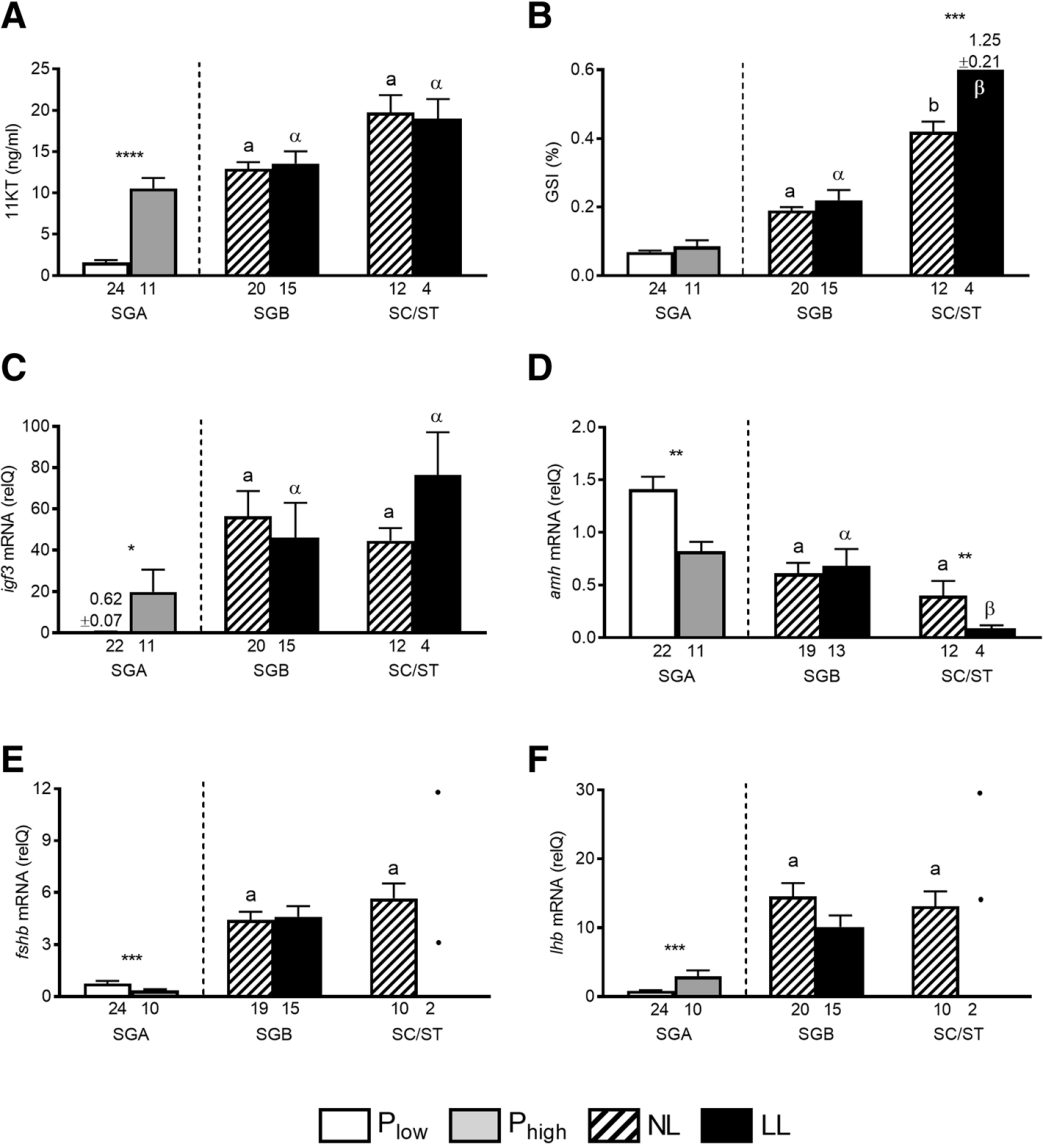
Reproductive parameters in male Atlantic salmon (experiment 2) exposed to normal light (NL) or to continuous additional light (LL). Based on testicular proliferation activity (see Fig. 4) and independent of the photoperiod, individuals showing type A spermatogonia (SGA) as the furthest developed germ cell type were assigned to groups with a low or a high proliferation activity (P_low_ and P_high_, respectively). Within the further developed stages showing type B spermatogonia (SGB), or spermatocytes/spermatids (SC/ST), all males showed high proliferation activity, irrespective of the photoperiod. Plasma 11-ketotestosterone levels (11KT; **a**; ng/ml); GSI, gonadosomatic index (**b**); relative testicular mRNA levels of *insulin-like growth factor 3* (*igf3*, **c**) or *anti Müllerian hormone* (*amh*, **D**); relative pituitary mRNA levels of *fshb* (**e**) or *lhb* (**f**). The number of individuals analyzed per group is given under the respective bars, showing means and SEM. In the SC/ST stage in (**e**) and (**f**), statistical comparison has not been included due to the small sample size and the individual values are shown. Statistical differences within a given graph between males falling into the same treatment group but showing different spermatogenic stages are indicated by different lower case Latin or Greek letters (*p* < 0.05; one way ANOVA followed by SNK post hoc test). Statistical differences within a given graph between recruited fish showing elevated proliferation activity (P_high_) or non-recruited fish showing low proliferation activity (P_low_) and between NL- or LL-exposed fish in the same stage of spermatogenic development are indicated by asterisks (Student t-test; *, *p* < 0.05; **, *p* < 0.01; ***, *p* < 0.001); the absence of an asterisk indicates the absence of statistically significant differences

Males in the SGA stage showing low proliferation activity also showed low (Fig. 5a; ~ 2 ng/ml) 11KT plasma levels. On the contrary, males with elevated proliferation activity showed 6-fold higher (~ 12 ng/ml) 11KT plasma levels. Males that had progressed to type B spermatogonia or beyond, showed similarly high 11KT plasma levels, irrespective of the photoperiod regime (Fig. 5a).

In NL-exposed males, the GSI doubled from levels below 0.1 to ~ 0.2 with the appearance of type B spermatogonia and increased further to 0.4 and higher when spermatocytes/spermatids were found (Fig. 5b). Elevated proliferation activity during the SGA stage was not reflected in significant changes of GSI values. Also, there was no significant difference between the GSI values in the NL and LL groups when type B spermatogonia were present. However, the LL group showed higher GSI values than the NL group in the SC/ST stage.

Testicular *igf3* transcript levels showed a pattern quite similar to 11KT and reliably reflected recruitment into maturation (Fig. 5c). Basal levels were recorded in males in the SGA stage with low 11KT levels and low proliferation activity, while elevated *igf3* transcript and 11KT plasma levels were found in males where spermatogonial and Sertoli cell proliferation had started. Elevated levels of *igf3* transcript were maintained in all males showing type B spermatogonia or further developed stages and no differences were found among maturing males exposed to different photoperiod regimes (Fig. 5c).

**Fig. 6:**
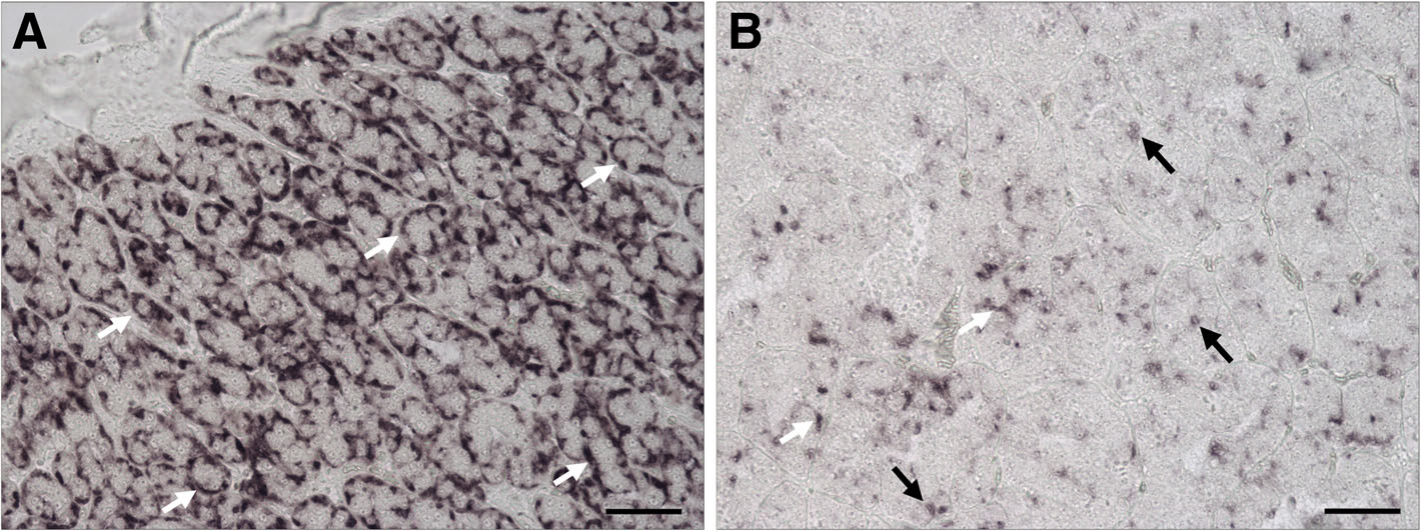
Five μm thick paraffin sections from salmon testis, showing the detection of *amh* mRNA in Sertoli cells. **a** In immature testis tissue, the cytoplasmic extensions of most Sertoli cells are strongly positive for *amh* mRNA (white arrows), enveloping unlabeled type A spermatogonia. **b** In early maturing testes containing type A and type B spermatogonia, *amh* mRNA is still detectable in the cytoplasm of Sertoli cells, some cells showing a strong staining (white arrows) that, however, no longer extends into the cytoplasmic extensions, while many Sertoli cells show a weak staining (black arrows), often restricted to the perinuclear area, or no staining at all. Please refer to Additional file 2: Figure S2 for a sense control staining. Black bars correspond to 50 μm length

The pattern of changes in testicular *amh* transcript levels were inverted compared to those of *igf3* (Fig. 5d). High levels were found in immature fish in the SGA stage, which decreased in males in the SGA stage showing elevated 11KT levels and proliferation activity. Compared to this recruitment-related decrease, the *amh* transcript levels did not decrease further in testis samples containing type B spermatogonia, and were similar in both, NL- and LL-exposed males. However, in testis tissue where spermatogenesis had progressed into meiosis/spermiogenesis, *amh* transcript levels decreased further and were 4-fold lower in the LL than in the NL group, the former also showing 3-fold higher GSI values (Fig. 5b, d). Since in zebrafish *amh* expression is localized to Sertoli cells [45, 54], which become increasingly outnumbered by germ cells during the development of spermatogenic cysts [33], it seems possible that part of the decrease of *amh* transcript levels reflects the dilution of *amh* expressing Sertoli cells by the increased mass of germ cells (viz. elevated GSI levels). Therefore, we examined the cellular localization of *amh* gene expression in testis tissue from LL-exposed males. Most Sertoli cells on sections from immature testis showed an intense staining for *amh* mRNA (Fig. 6a), where labeled cytoplasmic extensions of Sertoli cells enveloped unlabeled type A spermatogonia. On sections of maturing testis, some Sertoli cells still showed an intense labeling but it now appeared restricted in the perinuclear area and often showed a reduced intensity, while many Sertoli cells remained unlabeled (Fig. 6b). Germ cells and other tissue elements (e.g. blood vessels, blood cells, connective tissue elements) always remained unlabeled. Sections from immature males incubated with control (sense) probes remained unlabeled (Additional file 2: Figure S2 A and B).

The pituitary *fshb* and *lhb* transcript patterns resembled those of *igf3*: low levels were characteristic of males in the SGA stage with low proliferation activity and 11KT plasma concentrations while elevated levels were typically present in recruited males in the SGA stage and in the further advanced stages. No significant differences in gonadotropin gene expression were found between NL- and LL-exposed maturing males in the SGB stage (Figs. 5e, f).

**Fig. 7:**
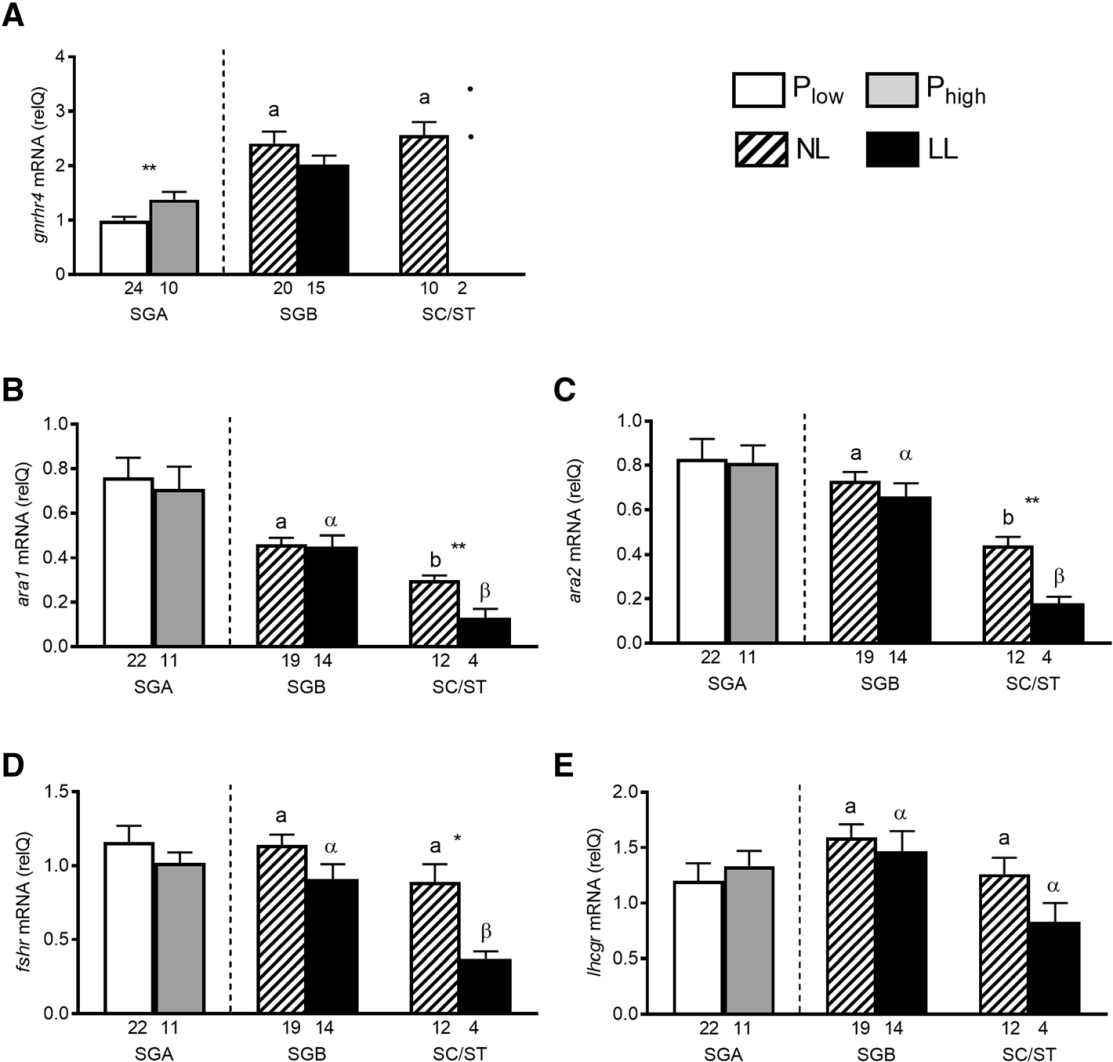
Reproductive hormone receptor gene expression in male Atlantic salmon (experiment 2) exposed to normal light (NL) or to continuous additional light (LL). Based on testicular proliferation activity (see Fig. 4) and independent of the photoperiod, individuals showing type A spermatogonia (SGA) as the furthest developed germ cell type were assigned to groups with a low or a high proliferation activity (P_low_ and P_high_, respectively). Within the further developed stages showing type B spermatogonia (SGB), or spermatocytes/spermatids (SC/ST), all males showed high proliferation activity, irrespective of the photoperiod. **a** Pituitary *gnrhr4* mRNA levels; **b** testicular *ara1* and **c** *ara2* mRNA levels; **d** testicular *fshr* and **e** *lhcgr* mRNA levels. The number of individuals analyzed per group is given under the respective bars, showing means and SEM. In the LL group in the SC/ST stage in **a** statistical comparison has not been included due to the small sample size and the individual values are shown. Statistical differences within a given graph between recruited fish showing elevated proliferation activity (P_high_) or non-recruited fish showing low proliferation activity (P_low_) and between NL- or LL-exposed fish in the same stage of spermatogenic development are indicated by asterisks (Student t-test; *, *p* < 0.05; **, *p* < 0.01); the absence of an asterisk indicates the absence of statistically significant differences.

Pituitary *gnrhr4* transcript levels increased significantly with the recruitment into maturation in SGA stage males (Fig. 7a). When comparing fish maturing under NL or LL conditions, pituitary *gnrhr4* transcript levels did not show differences in the SGB stage (Fig. 7a). Overall, the changes in *gnrhr4* transcript levels were moderate and much less pronounced than those recorded for gonadotropin transcript levels, and except for the up-regulation accompanying recruitment into pubertal maturation, did not show further changes related to the progress through spermatogenesis.

All males in the SGA stage indiscriminately showed clearly detectable testicular transcript levels of all hormone receptors (Fig. 7b-e). However, when GSI levels increased clearly with spermatogenesis reaching meiosis and in particular spermiogenesis, hormone receptor transcripts decreased, with the exception of *lhcgr* (Fig. 7b-e).

## Discussion

Significant changes of several parameters (pituitary Gnrh receptor and gonadotropin as well as testicular growth factor transcripts, plasma androgens, proliferation activity) accompanied the initiation of puberty in male Atlantic salmon. These changes took place without noteworthy alterations in testicular gonadotropin or androgen receptor transcript levels. Gonadotropin or androgen treatment of immature rainbow trout testis tissue changed gene expression patterns [25], indicating the receptors expressed in immature salmonid testis tissue are functional. We conclude that changes in the transcript levels of the receptors for the key-hormones regulating testis physiology (gonadotropins and androgens), are not relevant for the entry into puberty in Atlantic salmon. The receptors are already present and ready to respond to their ligands.

A hallmark feature of male puberty is the initiation of spermatogenesis, which is reflected in an increased single cell proliferation activity. Similar observations were reported in Atlantic salmon postsmolts at the verge of puberty [43], and in “grilsing” males, entering puberty after their first winter in seawater [55]. In these and the present study, the elevated proliferation activity of type A spermatogonia and Sertoli cells was associated with increases of pituitary *fshb* and *lhb* transcript and circulating androgen levels. Moreover, in the present study, we also found increased testicular *igf3* transcript levels. In previous studies in Atlantic salmon parr [40] but also in other species (rainbow trout: [56]; sea bass: [57]), the presence of type B spermatogonia was used as indicator of recruitment into puberty. However, this cell type occurred in our studies up to 3 months later than the increase in single cell proliferation activity. The strong association between the increase in proliferation activity, pituitary *fshb*, testicular *igf3* and plasma 11KT levels makes each one of these four parameters suitable to flag entry into puberty in male Atlantic salmon.

What signal(s) trigger(s) testis maturation? Hypophysectomy in eel stopped spermatogenesis that was re-started by gonadotropin injections [14, 58]. Genetic experiments showed that removing Fsh and Lh signaling blocked zebrafish spermatogenesis [15]. There is a certain overlap in the biological activities of the two gonadotropins when it comes to regulating testis physiology in zebrafish (e.g [19]). The overlap is related to a partial cross-activation of the Fshr by Lh [20] and to Fshr expression also by Leydig cells [21]. In salmonid fish, however, plasma Lh levels remain undetectable or very low until the males approach the spawning season [37, 59, 60]. Furthermore Fsh is the only gonadotropin present in the blood and released in response to Gnrh by the pituitary at the beginning of puberty in coho salmon [61]. Therefore, we consider Fsh as the primary signal to initiate salmon testis maturation. In precociously maturing male Atlantic salmon parr of less than 100 g body weight that mature in freshwater before smoltifying [40], in Atlantic salmon maturing directly after smoltification as 1 year-old fish with a body weight 150–350 g [43], and in the present study with 10-fold larger males, alike observations were made concerning the elevation of pituitary *fshb* transcript levels and the initiation of spermatogenesis. Independent of the astonishing plasticity concerning the timing of puberty, pituitary *fshb* transcript levels increase associated with the initiation of spermatogenic activity. FSH stimulated the proliferation of spermatogonia also in primates [9], rodents [6] and amphibians [10]. Taken together, in fish Fsh regulates Sertoli as well as Leydig cell activities [21–23], resulting in a comprehensive stimulation of spermatogenic activity by both growth factors [12] and androgens [26, 62]. Unfortunately, we did not have the possibility to quantify circulating Fsh, but elevated 11KT plasma and testicular *igf3* transcript levels in early pubertal salmon can be understood as reflecting increased Fsh stimulation.

Tissue culture studies using juvenile eel testes showed that androgen-induced down-regulation of *amh*, a Tgf-beta family member expressed by Sertoli cells, was a step sufficient to start spermatogenesis in this species [63]. In adult zebrafish, *amh* transcript levels were down-regulated by Fsh but not by androgens [44], and recent work showed that Amh inhibited zebrafish spermatogenesis via multiple mechanisms [45]. Therefore, an important aspect of the biological activity of Fsh in stimulating fish spermatogenesis is to down-regulate *amh* expression directly or via androgens. We confirm here for the Atlantic salmon that Sertoli cells express *amh* and that on the organ level, down-regulation of *amh* expression accompanies the start of puberty. Similar observations have been reported earlier in Atlantic salmon parr [41], rainbow trout [24] and sea bass [57]. Our in situ hybridization results suggest that different factors contribute to this decrease in *amh* transcript levels. One factor is the changing cellular composition of testis tissue in annually reproducing salmonids. Sertoli cells become increasingly outnumbered by germ cells during the progress of spermatogenesis, leading to a dilution of Sertoli by germ cell transcripts. In immature testes most Sertoli cells were strongly labeled for *amh* mRNA, while in testes displaying increased proliferation activity, some Sertoli cells still were intensely stained, others were less intensely stained, and some were not stained. This observation suggests that in many Sertoli cells of testis recruited into maturation, *amh* transcript levels were indeed down-regulated. It appears that *amh* transcript levels differ between Sertoli cells contacting different germ cell clones, potentially reflecting that *amh* mRNA levels change according to the stage of development and/or activity of a given spermatogenic cyst. We assume that locally different *amh* transcript levels result in locally different Amh protein levels. At sites with high levels of Amh, self-renewal proliferation of type A spermatogonia and hence the production of new spermatogenic cysts may prevail [44], facilitated by reduced Leydig cell activity and reduced Sertoli cell Igf3 release [45]. At sites of lower Amh levels, on the other hand, one would expect the opposite to occur: androgens and Igf3 would promote the differentiating proliferation of spermatogonia. Hence, Fsh stimulation would not only rely on its steroidogenic activity but also on decreased *amh* [44] and increased Igf3 transcript levels to stimulate spermatogenesis [11, 12, 48, 64]. It appears that the response to Fsh can differ locally and that not all spermatogenic cysts/Sertoli cells respond in the same manner at a given time.

What is responsible for an elevated release of Fsh at the beginning of testis maturation? Previous work in coho salmon showed that pituitary tissue from prepubertal fish responded to Gnrh by releasing Fsh, but not Lh [61]. Our work on Atlantic salmon postsmolts [43] and the present results in grilse showed that sorting males into two groups based on testicular proliferation activity, revealed increased Gnrh receptor expression in fish that started pubertal development. Photoperiod cues are major environmental signals modulating the activity of the brain-pituitary-gonad axis in fish [1, 65], and experimental photoperiods can inhibit or delay puberty in different species [66–68]. In experiment 2, all males exposed to NL were progressively recruited into maturation up until the beginning of June, and superallometric testis growth in NL-exposed fish in experiment 1 started after the Winter solstice. Therefore, we assume that yet uncharacterized, central nervous processes convey the increasing daylength to neuroendocrine centers in the salmon brain that trigger Fsh release. Given the Gnrh sensitivity of pituitaries from immature male coho salmon [61], and the increased *gnrhr4* transcript levels in recruited males in the present study, it is possible that the gradual increase in daylength following the winter solstice induced Gnrh release in an increasing fraction of the males. The ensuing Fsh release would then promote testis maturation by changing sex steroid and growth factor production.

The sudden exposure to continuous light, on the other hand, split the males into two response groups. About 40% of the males remained immature after exposure to LL. This percentage was similar to the percentage of fish not recruited yet in the NL group in February when exposure to LL started. In accordance with previous work in female Atlantic salmon [69], one possibility to understand this situation is to assume that for males that were not recruited yet when exposed to LL, the sudden increase in daylength closed the maturational window for this season and prevented entry into puberty. This would also suggest that an elevated release of Gnrh/Fsh did not occur. Also, in Atlantic salmon females 40% of LL-exposed fish remained immature, associated with very low pituitary *fshb* transcript and plasma E_2_ levels [42]. Interestingly, in males that had been recruited already, the switch to LL did not interrupt the already started maturation, suggesting that the type of response to LL depends on the physiological status of the individual male. Considering the higher GSI levels found under LL conditions in the SC/ST stage (Fig. 5b), we do not interpret this as an LL-mediated stimulation of testis growth but rather as a reflection of the fact that all maturing males in this group had started maturation before Feb 1 and continued testis development after LL started, while in the NL group, the start of pubertal testis growth was observed as late as mid-April. This scattered recruitment left less time for testis growth in males recruited later than Feb 1, resulting in a lower average GSI.

One of the biological activities of Fsh is to stimulate testicular androgen production [70]. The potent steroidogenic activity of Fsh in fish is related to the expression of *fshr* by piscine Leydig cells [21–23]. As discussed above, the elevated androgen levels at the beginning of maturation probably reflect Fsh bioactivity and exert feedback effects on the pituitary. Androgens are likely to be responsible for the observed increases in pituitary *lhb* transcript levels in recruited fish, involving aromatizable and 11-oxygenated androgens [71]. The situation is less clear regarding *fshb*. Sex steroids have a limited or no effect on *fshb* transcript levels in gonad-intact coho salmon [59], while for castrated Atlantic salmon parr following long-term steroid replacement, both positive and negative androgen feedback effects were reported on Fsh protein concentrations, depending on the experimental situation [72]. Also, a combined stimulatory effect of Igf1 and Gnrh has been described for coho salmon pituitary cells in primary tissue culture [73]. Clearly, more work is required regarding the neuroendocrine or gonadal feedback regulation of *fshb* expression.

Considering the androgen plasma levels over the complete reproductive cycle, we can differentiate three steps of up-regulation. The first one concurred with the activation of spermatogonial and Sertoli cell proliferation when plasma androgens increased to levels of ~ 10 ng/ml. This level was maintained until reaching meiosis/spermiogenesis when a second increase to 20–30 ng/ml was recorded. Maximum GSI levels were attained in the presence of 11KT or T levels not surpassing ~ 30 ng/ml. When testis weight started to decline due to the progress of spermiogenesis while the production of spermatogenic cysts had stopped, the third and last up-regulation of in particular 11KT levels occurred, reaching levels of up to 100 ng/ml. Evidently, this final increase is unrelated to the largely completed spermatogenic process. Instead, it may be relevant to support the full development of secondary sexual characteristics and male reproductive behavior, representing androgen functions outside the testis.

A prominent mediator of the biological activity of androgens is the nuclear androgen receptor. In mammals, it is expressed by different somatic cell types in testis tissue (Sertoli cells, Leydig cells and myoid cells), but not by germ cells [32]. Loss of the androgen receptor only in Sertoli cells was sufficient to block spermatogenesis at mid-meiosis in mice [29]. Remarkably, loss of the nuclear androgen receptor function in zebrafish did not block spermatogenesis since mutant testis tissue still contained all germ cell types including spermatozoa; also, mutant sperm was able to fertilize wild-type eggs in vitro. However, the testis size in mutants was only a fifth of the size in wild-type males [62, 74]. Hence, sperm production does not strictly depend on androgen signaling in zebrafish. Other signaling pathways, such as growth factor signaling (see above) or the direct stimulation of spermiogenesis by Lh [75], may compensate in part for the loss of *ar* function in zebrafish. Still, androgens clearly stimulated spermatogenesis in different fishes, indicating that an important function of Fsh-stimulated androgen production is to promote the production of new spermatogenic cysts and to drive them into differentiating proliferation, such as described in eel or zebrafish [26, 27, 76].

Two androgen receptors were identified in the Atlantic salmon [48], both belonging to the Ara branch [77]. There is no information on the cellular sites of androgen receptor expression in the salmon testis. Our trials to specifically localize *ara1/ara2* expression in testis tissue by in situ hybridization were not successful. In zebrafish, showing a single *ar* gene, Sertoli cells associated with type A spermatogonia expressed *ar* mRNA [78]. The main changes (down-regulation) in testicular *ara1/2* transcript levels occurred when GSI levels increased clearly upon reaching meiosis/spermiogenesis in both experiments. A study examining testicular *ar* transcript levels during a maturational cycle in eel showed akin results: high transcript levels were present when the testes were small and contained type A spermatogonia, but decreased significantly and remained at a low level after spermatogenesis was activated and testis weight increased [79]. These results may reflect the fact that the somatic cells expressing *ar* become increasingly outnumbered, and hence their mRNAs increasingly diluted, by the geometrically increasing germ cell number in differentiating spermatogenic cysts. Evidently, the changing tissue composition complicates the understanding of transcript level data, when transcripts are present in a specific cell type that changes in number. The gonadotropin receptor transcript levels showed patterns quite similar to those of the androgen receptors and may therefore also reflect the increasing abundance of cells not expressing gonadotropin receptor transcripts. Also in rainbow trout [38, 39] and sea bass [36], the initiation of puberty occurred without changes in these transcripts.

## Conclusions

Testicular transcript levels of gonadotropin and androgen receptors are clearly detectable and stable during the start of pubertal testis development in Atlantic salmon. However, changes in the availability of the ligands for these receptors are relevant for triggering testis maturation. Early morphological and molecular signs for puberty are increases in the proliferation of type A spermatogonia and Sertoli cells and elevated *igf3* transcript levels. While a certain increase in the pituitary sensitivity to Gnrh may be important for the start of puberty, the regulation of Gnrh release and its modulation by environmental factors (photoperiod) seem to be of decisive relevance for the timing of initiating testis maturation.

## Additional files

Additional file 1: **Figure S1.** Relative pituitary *fshb* mRNA (top row) and 11KT plasma levels (lower row) of Atlantic salmon males showing either type A_und_ or type A_diff_ spermatogonia as furthest developed germ cell type, sampled before or after the Winter solstice. Individual values, means and standard error of the mean are shown. The number of individuals analyzed per group is given under the respective groups. Means were compared by Student t-test. (TIF 248 kb)

Additional file 2: **Figure S2.** Testis sections from an immature male showing type A spermatogonia as furthest developed germ cell stage after incubation with sense control (**A**) or antisense cRNA probe (**B**) recognizing *amh* mRNA. Specific staining of the Sertoli cell cytoplasm is only seen with the antisense cRNA probe. (TIF 2650 kb)

Additional file 3: **Table S1.** Furthest developed germ cell stage found during the different sampling dates of experiment 1. Column abbreviations represent date, number of males, or germ cell stages (A_und_ – type A undifferentiated spermatogonia; A_diff_ – type A differentiating spermatogonia; B – type B spermatogonia; SC/ST – spermatocytes and/or spermatids; SZ – spermatozoa). The vertical line indicates transfer from seawater to freshwater; shading of the dates indicates that 50% (Oct 12) or all showed running milt. (DOCX 22 kb)

Additional file 4: **Table S2.** Percent distribution of males showing type A spermatogonia (SGA), type B spermatogonia (SGB), or spermatocytes/spermatids (SC/ST) as furthest developed germ cell stage on the different sampling dates of experiment 2. After the initial control sampling on January 8, exposure to normal light (NL) continued, or exposure to continuous light (LL) started on February 1 for half of the animals. (DOCX 20 kb)

## Data Availability

The datasets used and/or analysed during the current study are available from the corresponding author on reasonable request.

## Abbreviations

11KT: 11-ketotestosterone
A_diff_: Differentiating type A spermatogonia
AMH: Anti-Mullerian hormone
ARA1/2: Androgen receptor type a 1 or 2
A_und_: Undifferentiated type A spermatogonia
EF1α: Elongation factor 1α
ELISA: Enzyme-linked immunosorbent assay
FSH: Follicle-stimulating hormone
FSHB: Follicle-stimulating hormone beta subunit
FSHR: Follicle-stimulating hormone receptor
GNRH: Gonadotropin-releasing hormone
GNRHR4: Gonadotropin-releasing hormone receptor type 4
GSI: Gonado-somatic index
IGF3: Insulin-like growth factor 3
ISH: In situ hybridization
LH: Luteinizing hormone
LHB: Luteinizing hormone beta subunit
LHCGR: Luteinizing hormone chorionic gonadotropin receptor
LL: Continuous light photoperiod
NARA: Norwegian Animal Research Authority
NL: Natural light photoperiod
PCR: Polymerase chain reaction
pH 3: Phosphorylated histone H3
SC: Spermatocytes
SGA: Spermatogonia A
SGB: Spermatogonia B
SNK: Student newman keuls test
ST: Spermatids
SZ: Spermatozoa
T: Testosterone
TGF: Transforming growth factor
