# Correction to: Entry into puberty is reflected in changes in hormone production but not in testicular receptor expression in Atlantic salmon (*Salmo salar*)

**DOI:** 10.1186/s12958-019-0503-x

**Published:** 2019-07-16

**Authors:** Rüdiger W. Schulz, Geir Lasse Taranger, Jan Bogerd, Wouter Nijenhuis, Birgitta Norberg, Rune Male, Eva Andersson

**Affiliations:** 1 Research Group Reproduction and Developmental Biology, Institute of Marine Research, P.O.Box 1870, Nordnes, 5817 Bergen, Norway; 2 Reproductive Biology Group, Division Developmental Biology, Department Biology, Science Faculty, Utrecht University, Utrecht, The Netherlands; 3 Department of Biological Sciences, University of Bergen, Bergen, Norway

**Correction to: Reprod Biol Endocrinol**

**https://doi.org/10.1186/s12958-019-0493-8**

Following publication of the original article [1], the authors would like to apologize for an error in Fig. 5e, the correct graph is presented below and shows the significant increase in pituitary mRNA levels of *fshb* in recruited males in the SGA stage.

**Fig. 5:**
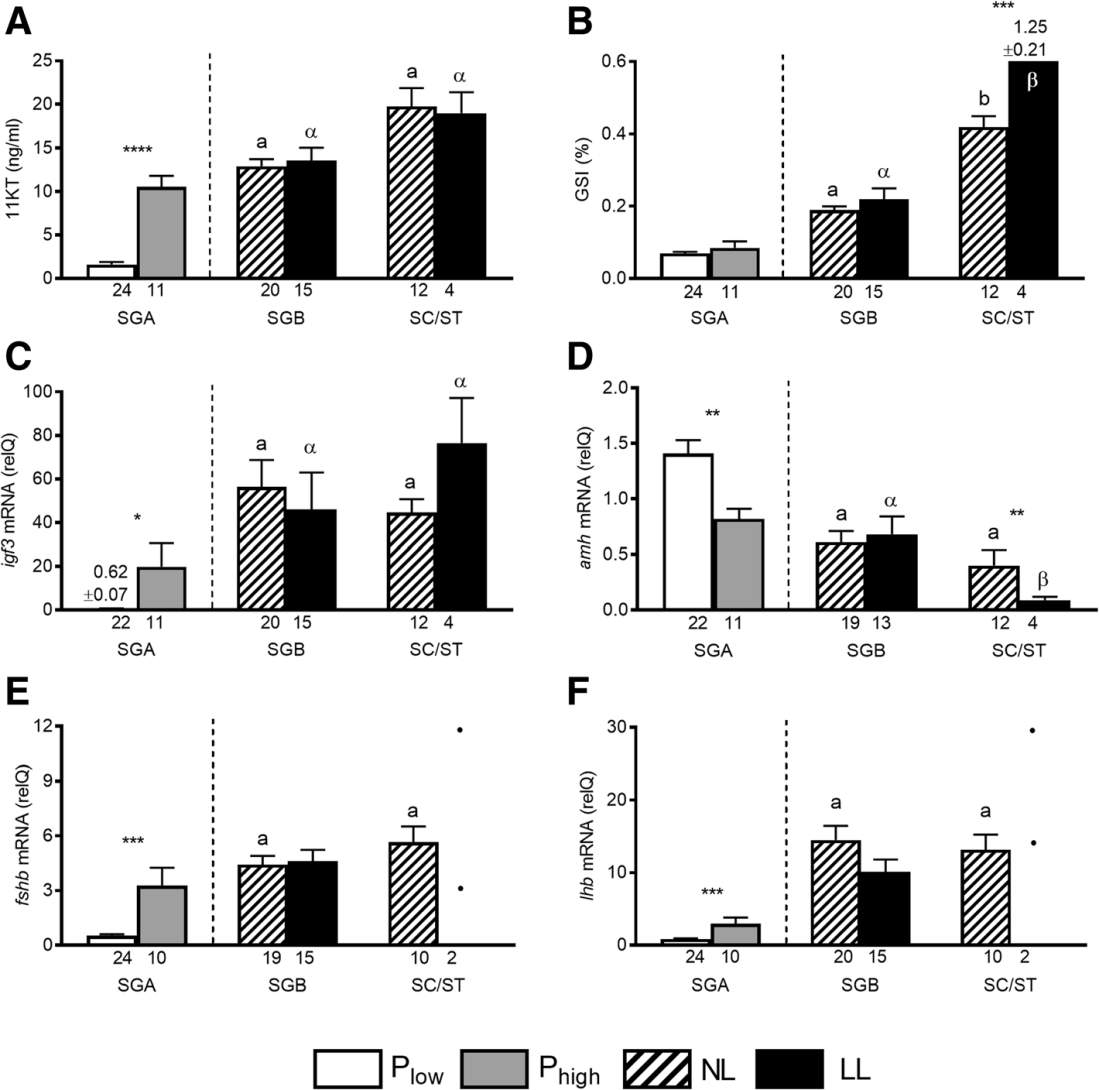
Reproductive parameters in male Atlantic salmon (experiment 2) exposed to normal light (NL) or to continuous additional light (LL). Based on testicular proliferation activity (see Fig. 4) and independent of the photoperiod, individuals showing type A spermatogonia (SGA) as the furthest developed germ cell type were assigned to groups with a low or a high proliferation activity (P_low_ and P_high_, respectively). Within the further developed stages showing type B spermatogonia (SGB), or spermatocytes/spermatids (SC/ST), all males showed high proliferation activity, irrespective of the photoperiod. Plasma 11-ketotestosterone levels (11KT; **a**; ng/ml); GSI, gonadosomatic index (**b**); relative testicular mRNA levels of *insulin-like growth factor 3* (*igf3*, **c**) or *anti Müllerian hormone* (*amh*, **d**); relative pituitary mRNA levels of *fshb* (**e**) or *lhb* (**f**). The number of individuals analyzed per group is given under the respective bars, showing means and SEM. In the SC/ST stage in e and f, statistical comparison has not been included due to the small sample size and the individual values are shown. Statistical differences within a given graph between males falling into the same treatment group but showing different spermatogenic stages are indicated by different lower case Latin or Greek letters (*p* < 0.05; one way ANOVA followed by SNK post hoc test). Statistical differences within a given graph between recruited fish showing elevated proliferation activity (P_high_) or non-recruited fish showing low proliferation activity (P_low_) and between NL- or LL-exposed fish in the same stage of spermatogenic development are indicated by asterisks (Student t-test; *, *p* < 0.05; **, *p* < 0.01; ***, *p* < 0.001); the absence of an asterisk indicates the absence of statistically significant differences

The authors regret any inconvenience caused.

## References

[1] Schulz RW, Taranger GL, Bogerd J, Nijenhuis W, Norberg B, Male R, Andersson E. Entry into puberty is reflected in changes in hormone production but not in testicular receptor expression in Atlantic salmon (*Salmo salar*). Reprod Endocrinol Biol. 2019;17(48) 10.1186/s12958-019-0493-8. 10.1186/s12958-019-0493-8 PMC6588918 31226998

