# Supplementary figures and images for: Entry into puberty is reflected in changes in hormone production but not in testicular receptor expression in Atlantic salmon (Salmo salar)
Source: Reprod Biol Endocrinol. 2019 Jun 21;17:48. doi: 10.1186/s12958-019-0493-8 (PMC6588918; doi:10.1186/s12958-019-0493-8)

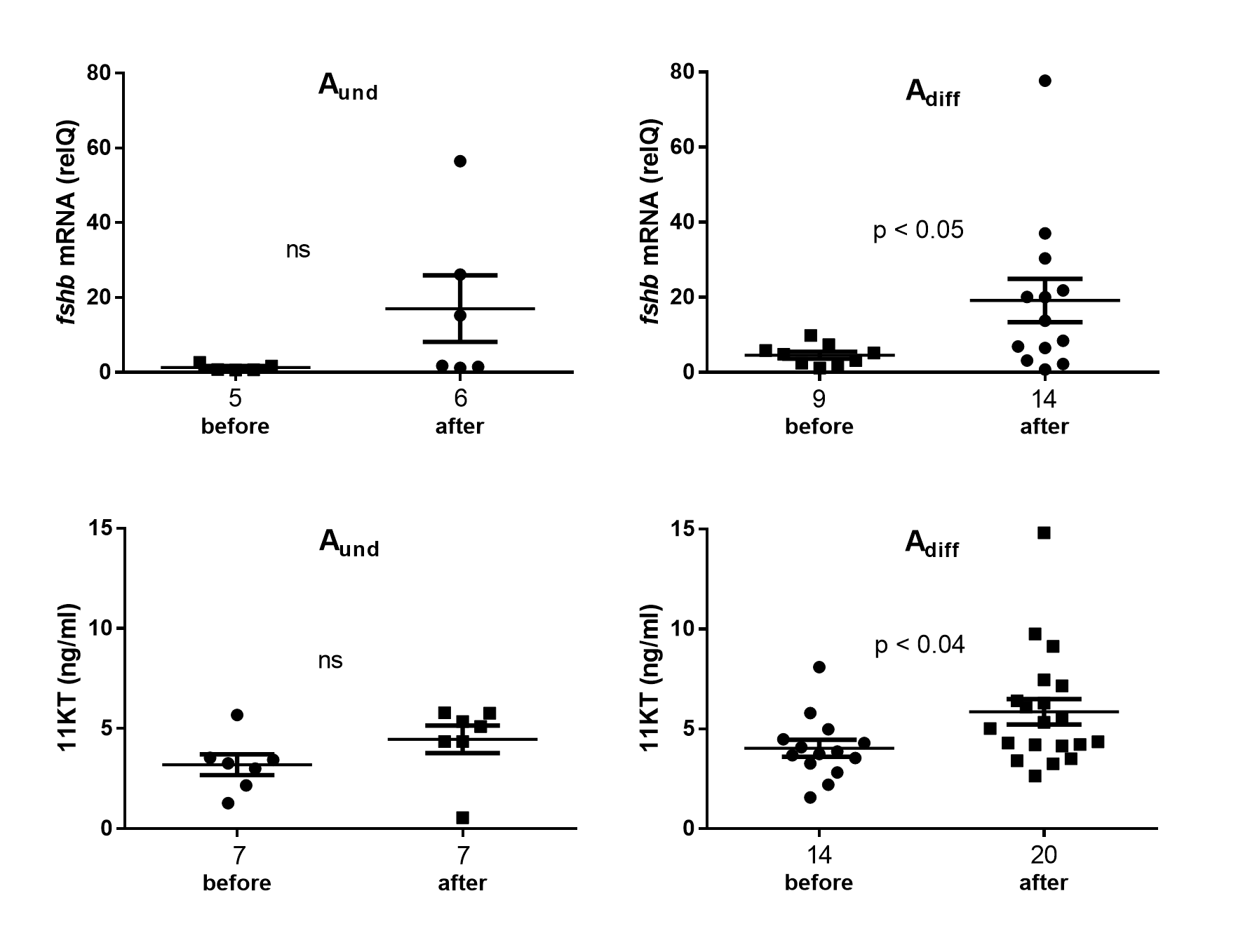

Supplement: Supplementary file 1 — Figure S1. Relative pituitary fshb mRNA (top row) and 11KT plasma levels (lower row) of Atlantic salmon males showing either type Aund or type Adiff spermatogonia as furthest developed germ cell type, sampled before or after the Winter solstice. Individual values, means and standard error of the mean are shown. The number of individuals analyzed per group is given under the respective groups. Means were compared by Student t-test. (TIF 248 kb) [file 12958_2019_493_MOESM1_ESM.tif]

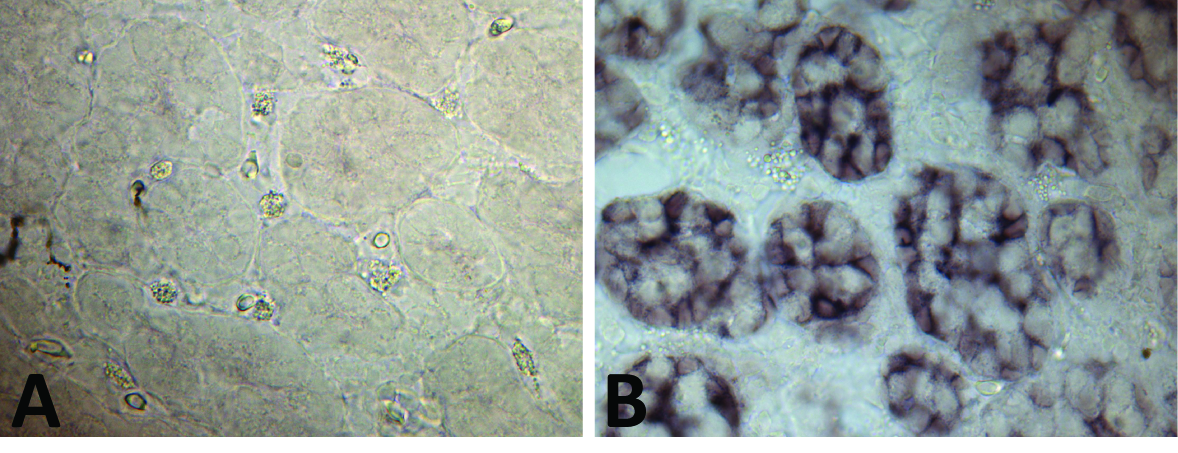

Supplement: Supplementary file 2 — Figure S2. Testis sections from an immature male showing type A spermatogonia as furthest developed germ cell stage after incubation with sense control (A) or antisense cRNA probe (B) recognizing amh mRNA. Specific staining of the Sertoli cell cytoplasm is only seen with the antisense cRNA probe. (TIF 2650 kb) [file 12958_2019_493_MOESM2_ESM.tif]
