# Supplementary material for: Entry into puberty is reflected in changes in hormone production but not in testicular receptor expression in Atlantic salmon (Salmo salar)
Source: Reprod Biol Endocrinol. 2019 Jun 21;17:48. doi: 10.1186/s12958-019-0493-8 (PMC6588918; doi:10.1186/s12958-019-0493-8)
Supplement: Supplementary file 3 — Table S1. Furthest developed germ cell stage found during the different sampling dates of experiment 1. Column abbreviations represent date, number of males, or germ cell stages (Aund – type A undifferentiated spermatogonia; Adiff – type A differentiating spermatogonia; B – type B spermatogonia; SC/ST – spermatocytes and/or spermatids; SZ – spermatozoa). The vertical line indicates transfer from seawater to freshwater; shading of the dates indicates that 50% (Oct 12) or all showed running milt. (DOCX 22 kb) [file 12958_2019_493_MOESM3_ESM.docx]

| date | n | Aund | Adiff | B | SC/ST | SZ |
| --- | --- | --- | --- | --- | --- | --- |
| 28-Aug | 6 |  |  |  |  |  |
| 10-Oct | 5 |  |  |  |  |  |
| 19-Nov | 8 |  |  |  |  |  |
| 8-Jan | 7 |  |  |  |  |  |
| 18-Feb | 8 |  |  |  |  |  |
| 10-Mar | 8 |  |  |  |  |  |
| 15-Apr | 8 |  |  |  |  |  |
| 12-May | 6 |  |  |  |  |  |
| 18-Jun | 7 |  |  |  |  |  |
| 7-Jul | 4 |  |  |  |  |  |
| 18-Aug | 5 |  |  |  |  |  |
| 14-Sep | 3 |  |  |  |  |  |
| 12-Oct | 4 |  |  |  |  |  |
| 16-Nov | 3 |  |  |  |  |  |
| 14-Dec | 2 |  |  |  |  |  |

**Table S1.** Furthest developed germ cell stage found during the different sampling dates of experiment 1. Column abbreviations represent date, number of males, or germ cell stages (A_und_ – type A undifferentiated spermatogonia; A_diff_ – type A differentiating spermatogonia; B – type B spermatogonia; SC/ST – spermatocytes and/or spermatids; SZ – spermatozoa). The vertical line indicates transfer from seawater to freshwater; shading of the dates indicates that 50% (Oct 12) or all showed running milt.
