# Supplementary material for: Entry into puberty is reflected in changes in hormone production but not in testicular receptor expression in Atlantic salmon (Salmo salar)
Source: Reprod Biol Endocrinol. 2019 Jun 21;17:48. doi: 10.1186/s12958-019-0493-8 (PMC6588918; doi:10.1186/s12958-019-0493-8)
Supplement: Supplementary file 4 — Table S2. Percent distribution of males showing type A spermatogonia (SGA), type B spermatogonia (SGB), or spermatocytes/spermatids (SC/ST) as furthest developed germ cell stage on the different sampling dates of experiment 2. After the initial control sampling on January 8, exposure to normal light (NL) continued, or exposure to continuous light (LL) started on February 1 for half of the animals. (DOCX 20 kb) [file 12958_2019_493_MOESM4_ESM.docx]

|  |  | NL |  |  | LL |  |
| --- | --- | --- | --- | --- | --- | --- |
|  | SGA | SGB | SC/ST | SGA | SGB | SC/ST |
| Jan 08 | 100 | - | - | - | - | - |
| Feb 18 | 45 | 55 | - | 40 | 60 | - |
| Mar 19 | 33 | 66 | - | 37 | 63 | - |
| Apr 25 | 11 | 89 | - | 45 | 55 | - |
| Jun 11 | - | - | 100/0 | 44 | - | 28/28 |

**Table S2.** Percent distribution of males showing type A spermatogonia (SGA), type B spermatogonia (SGB), or spermatocytes/spermatids (SC/ST) as furthest developed germ cell stage on the different sampling dates of experiment 2. After the initial control sampling on January 8, exposure to normal light (NL) continued, or exposure to constant light (LL) started on February 1 for half of the animals.
